# The New World Screwworm in the United States: A Narrative Review Anchored to the 2025 Travel-Associated Human Case

**DOI:** 10.7759/cureus.94039

**Published:** 2025-10-07

**Authors:** Kirubel T Hailu, Alousious Kasagga, Ryan R Haddad

**Affiliations:** 1 Public Health, University College Cork, Cork, IRL; 2 Pathology, Peking University, Beijing, CHN; 3 Clinical Research, California Institute of Behavioral Neurosciences and Psychology, Fairfield, USA

**Keywords:** climate change impact on health, cochliomyia hominivorax, disease surveillance, myiasis infestation, one-health, screwworm

## Abstract

The New World screwworm (*Cochliomyia hominivorax*) is an obligate parasitic fly whose larvae invade and consume living tissue, causing myiasis with severe consequences for human health, animal welfare, and agricultural productivity. Although eradicated from the United States in the 1960s through the sterile insect technique (SIT) and contained thereafter by a Panama-based biological barrier, the parasite remains endemic in parts of South America and the Caribbean. Endemicity in these regions sustains the risk of reintroduction into screwworm-free areas. A recent travel-associated case of human screwworm in the United States highlights this ongoing threat, though no evidence of local transmission has been detected. While the public health risk in the United States remains low, the agricultural stakes are substantial: screwworm outbreaks can cause significant morbidity and mortality in livestock, necessitating costly interventions and resulting in considerable economic losses if detection and control are delayed. This review synthesizes the biology and pathogenesis of *C. hominivorax*, the historical trajectory of eradication efforts, details of the most recent U.S. case, the ongoing risks to public health and agriculture, and current and emerging prevention strategies. We emphasize that future resilience will depend on embedding surveillance and control in a One Health framework, maintaining redundant SIT capacity, integrating climate-informed risk models, and fostering international collaboration. The recent U.S. case should be interpreted as a sentinel event that reaffirms the fragility of eradication gains and the need for sustained vigilance.

## Introduction and background

The New World screwworm fly (*Cochliomyia hominivorax*), a member of the Calliphoridae family, is a parasitic insect of profound medical, veterinary, and economic importance. Unlike most dipteran larvae, which preferentially colonize necrotic tissue, *C. hominivorax* larvae invade and consume living tissue of warm-blooded hosts, causing a rapidly progressive condition known as myiasis. This obligate parasitism can result in severe pain, secondary bacterial infections, disfigurement, and, in the absence of timely treatment, substantial morbidity or death in both humans and animals. In livestock, infestations lead to reduced productivity, increased veterinary costs, and mortality, thereby generating significant economic burdens. Historically, outbreaks have inflicted devastating consequences on the cattle industries of the Americas, with estimated losses in the United States exceeding hundreds of millions of dollars annually before eradication programs in the mid-20th century [1-3].

Through the pioneering use of the sterile insect technique (SIT), which suppresses target populations by releasing large numbers of factory-reared, sterilized males, because female *C. hominivorax* typically mate once, matings with sterile males yield no viable offspring, the United States successfully eliminated endemic screwworm populations by 1966, and subsequent regional collaborations extended eradication throughout Central America. Nonetheless, *C. hominivorax* remains endemic in parts of South America and the Caribbean, with recent resurgences documented in Central America. These areas represent a continual source of potential reintroduction, particularly given increasing global connectivity, cross-border livestock trade, and climate-driven changes in vector distribution [3-6].

Recent U.S. experience also includes the 2016-2017 Florida Keys outbreak in Key deer, underscoring that sporadic introductions can occur even with a functioning regional barrier. On August 4, 2025, the U.S. Centers for Disease Control and Prevention (CDC) confirmed the nation's first human case of travel-associated screwworm infestation in decades. The patient, a Maryland resident returning from El Salvador, presented with parasitic larvae embedded in living tissue, consistent with *C. hominivorax*. Although the infection was successfully treated and epidemiologic investigations found no evidence of local transmission, the case is of considerable significance. It coincides with an ongoing regional outbreak in Central America, underscoring the persistent vulnerability of U.S. public health and agriculture to transboundary reintroduction events. In response, the U.S. Department of Agriculture (USDA) launched intensified surveillance across Maryland, Virginia, and the District of Columbia, and announced the establishment of a new sterile screwworm production facility in Texas as a precautionary measure [7-9].

This narrative review synthesizes the historical trajectory, current epidemiological context, and future challenges associated with *C. hominivorax*. Specifically, we 1) review the biology and clinical pathology of screwworm infestations, 2) summarize past eradication campaigns and the 2025 U.S. case, 3) assess public health, veterinary, and economic risks associated with reemergence, and 4) evaluate current and emerging strategies for surveillance and control within a One Health framework. By situating the recent Maryland case within the broader ecological and epidemiological landscape, we highlight the continued need for integrated vigilance to safeguard human, animal, and agricultural health.

## Review

Methods

This article is presented as a narrative review, synthesizing biological, historical, and contemporary evidence related to the New World screwworm. While it does not employ the exhaustive, quantitative approach of a systematic review, the selection of sources was guided by a comprehensive and rigorous search to provide a robust and integrative overview of the topic. Our search included a broad range of authoritative sources, with a preference for peer-reviewed journal articles, authoritative government reports, and technical documents from international organizations. Sources were identified through comprehensive searches of PubMed, Google Scholar, and official agency websites, including the U.S. CDC, USDA, Food and Agriculture Organization (FAO), and International Atomic Energy Agency (IAEA). When multiple sources covered the same event or program, we prioritized the most recent, most comprehensive, and primary agency reports. To reflect a One Health lens, we deliberately balanced human clinical, veterinary, environmental, and operations sources.

Specific search terms used were “Cochliomyia hominivorax”, “screwworm”, “myiasis”, “sterile insect technique”, “eradication”, “outbreak”, “Central America”, “United States”, and “One Health”. The search was restricted to English-language publications between 1950 and 2025, though seminal works on eradication predating this range were also included. Gray literature, such as press releases and news coverage, was considered only when providing critical details on the 2025 U.S. case that were not yet available in peer-reviewed sources. Data were extracted narratively rather than quantitatively. No quantitative pooling or meta-analysis was attempted due to heterogeneity in designs, outcomes, and aims, consistent with the scope of a descriptive synthesis. The overall objective was to provide a rigorous, integrative overview situated within a One Health framework.

Discussion

Biology and Pathogenesis

The New World screwworm (*C. hominivorax* Coquerel, 1858) is an obligate parasitic dipteran of the Calliphoridae family. Its life cycle is holometabolous, comprising egg, larva, pupa, and adult stages. Gravid females deposit clusters of 100-300 eggs at the margins of wounds, mucous membranes, or natural body orifices of warm-blooded vertebrates. Eggs hatch within 12-24 hours, releasing first-instar larvae that immediately penetrate healthy tissue. Over a period of four to seven days, larvae progress through three instars while feeding aggressively and enlarging the wound cavity. Mature larvae then exit the host and pupate in soil; adults typically emerge after 7-54 days, depending on temperature and humidity. Under warm conditions, the entire life cycle can be completed in approximately three to four weeks, enabling rapid population expansion in endemic regions [1,2,10]. The host range includes a broad spectrum of domestic and wild mammals, with livestock, particularly cattle, sheep, and goats, most frequently affected. Human cases are less common but clinically significant. Females are monogamous and typically mate only once, a trait critical to the success of SIT programs [10-12].

Pathogenesis is characterized by larvae consuming living tissue, a distinguishing feature from most blowflies. Mouth hooks and proteolytic secretions facilitate deep invasion. Lesions are painful, hemorrhagic, and prone to bacterial superinfection. In animals, infestations often localize at neonatal navels or surgical wounds; in humans, they affect cutaneous wounds and sometimes nasal, auditory, or ocular sites. Untreated cases may progress to muscle, cartilage, or bone destruction, with severe morbidity or mortality [1,2,13]. The parasite's short generation time, obligate parasitism, and capacity to colonize living tissue drive its invasive potential and historical economic losses.

Historical Perspective

**Figure 1:**
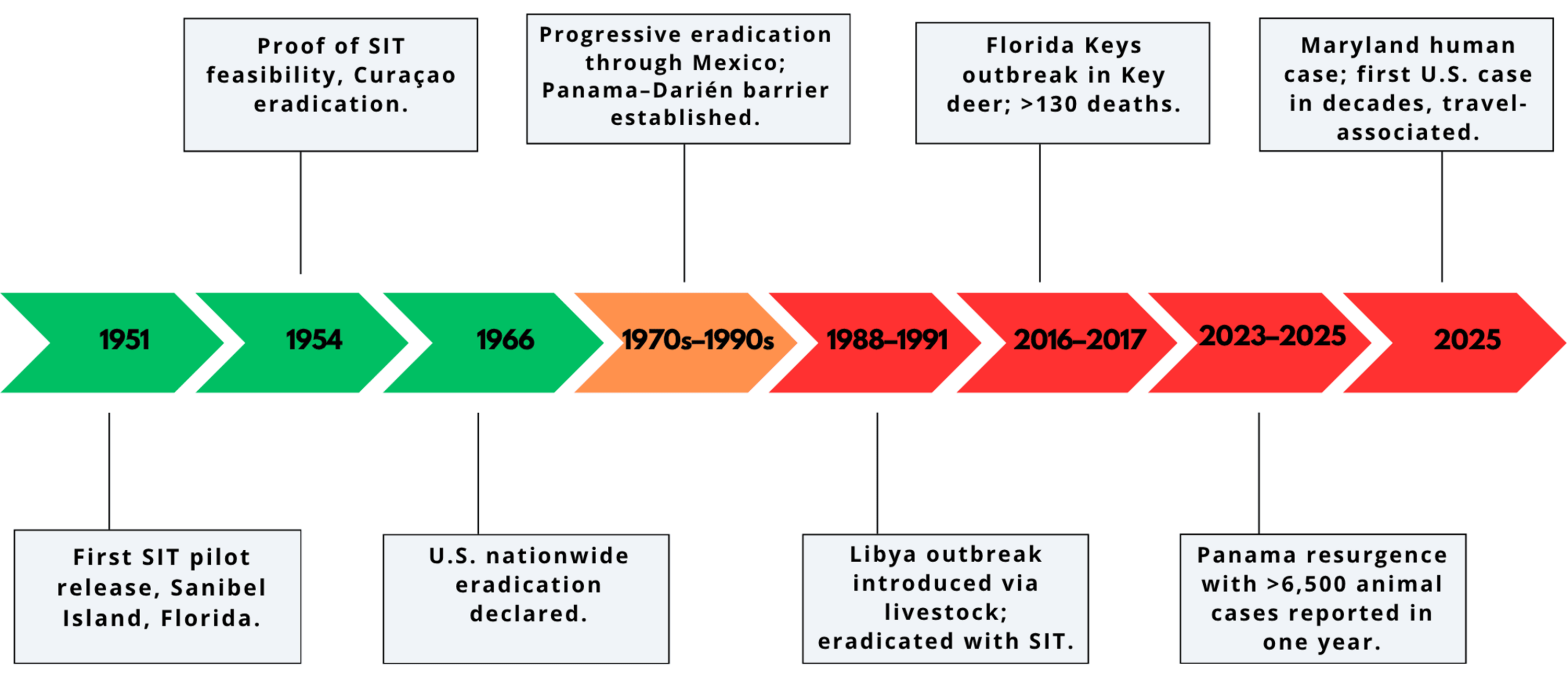
Timeline of screwworm eradication and major incursions (1951-2025) Events are color-coded to highlight eradication milestones (green), regional control campaigns (orange), and outbreaks/resurgences (red). Early SIT demonstrations in Florida (1951) and Curaçao (1954) led to nationwide eradication in the United States (1966). Regional eradication efforts advanced south through Mexico, culminating in the establishment of the Panama-Darién barrier in the 1990s. Major incursions include the Libya outbreak introduced via livestock (1988-1991), the Florida Keys outbreak in Key deer (>130 deaths, 2016-2017), the resurgence in Panama (2023-2025), and the 2025 Maryland case SIT: sterile insect technique Image credit: This is an original image created by the author Kirubel T. Hailu

Before its eradication, *C. hominivorax* was widely distributed across the Western Hemisphere, from the southern United States to northern Argentina. In the mid-20th century, infestations caused annual livestock losses exceeding USD $200 million in the United States alone [10,11]. Early control efforts, reliant on wound dressings and larvicides, were labor-intensive and unsustainable [2]. The paradigm shifted with the development of SIT by Knipling and Bushland in the 1950s. Pilot releases on Sanibel Island (1951) and Curacao (1954) proved that sterile males could eliminate local populations by exploiting female monogamy [2,12]. Subsequent campaigns eradicated screwworm from Florida by 1959 and from the entire United States by 1966, marking the first successful continental application of SIT [11]. Regional efforts extended the eradication southward through Mexico and Central America, culminating in the establishment of a permanent barrier in Panama in the 1990s [13]. The Pacora sterile-fly facility, releasing ~50 million sterile flies weekly, remains essential in preventing northward reinvasion from South America [3,13]. A summary of eradication milestones and major incursions is presented in Figure 1.

Despite these advances, *C. hominivorax* persists in South America and the Caribbean, with localized outbreaks documented in Brazil, Guyana, and Trinidad [14]. International spread has occurred before, most notably to Libya in 1988, where an outbreak introduced via livestock required coordinated eradication by 1991 [15]. Central America has experienced a rise in cases since 2023 [8].

The 2025 U.S. Case

On August 4, 2025, the CDC confirmed the first U.S. human screwworm case in decades: a Maryland resident who had recently returned from El Salvador. The patient presented with a painful, enlarging wound, and infestation with *C. hominivorax* was confirmed through larval identification. The individual was treated with surgical debridement and antiparasitic therapy, leading to full recovery [2,16].

Epidemiological investigation found no evidence of autochthonous transmission. The USDA and CDC conducted entomological surveillance within a 20-mile radius covering Maryland, Virginia, and Washington, D.C., and no screwworm flies were detected. The case was, therefore, classified as travel-associated [16]. This event coincided with active screwworm outbreaks in Central America, particularly El Salvador. In response, USDA announced plans for a new sterile-fly production facility in Texas, designed to provide domestic capacity for producing hundreds of millions of sterile males weekly, strengthening national preparedness against incursions. Comparative data on prior introductions and outbreaks in nonendemic regions are summarized in Table 1.

**Table 1:** Selected nonendemic introductions and travel-associated screwworm cases (1988-2025) Source: Data compiled from published case reports, USDA-APHIS [3,8], FAO/IAEA field reports [15], and CDC communications [16] FAO: Food and Agriculture Organization; IAEA: International Atomic Energy Agency; SIT: sterile insect technique; USDA: U.S. Department of Agriculture; APHIS: Animal and Plant Health Inspection Service; CDC: Centers for Disease Control and Prevention

Year	Location (nonendemic)	Travel origin/source	Host(s) affected	Outcome
1988-1991	Libya	Imported livestock (South America)	Cattle, sheep, goats, and camels	Major outbreak affecting ~1.3 million km²; eradicated by 1991 via FAO/IAEA-led SIT campaign
1998-2005	Europe: isolated imported human cases reported in travelers (United Kingdom, France, others)	Travelers returning from Latin America, Caribbean, and Africa	Human (cutaneous, aural, and nasal myiasis)	Isolated imported cases; no local transmission
2016-2017	Florida Keys, USA	Introduction source uncertain (likely Caribbean)	Wildlife (key deer), pets	Localized outbreak; >130 key deer fatalities; eradicated within months using SIT
2025	Maryland, USA	Traveler returning from El Salvador	Human (cutaneous myiasis)	First U.S. human case in decades; successfully treated; no evidence of transmission

Public Health and Agricultural Risks

Human screwworm myiasis is rare but clinically severe. Infestations cause rapidly enlarging, painful wounds that can progress to deeper tissues, with risks of secondary infection, sepsis, and mortality. Prompt recognition and removal of larvae are essential [14,17]. The Maryland case demonstrated low public-health risk domestically, with no onward transmission after surveillance [16]. Wildlife hosts, especially cervids, can sustain transmission and complicate detection, as illustrated by the Florida Keys event; therefore, integrated livestock-wildlife surveillance is essential.

Agricultural risks are much greater. Livestock infestations, if undetected, can expand rapidly, leading to high morbidity, mortality, and economic losses. Neonates are particularly vulnerable [18]. Outbreaks in Panama since 2023 have surged dramatically from ~25 cases annually to more than 6,500 in a single year [8]. Economic analyses project that an outbreak similar to the 1976 Texas event would, in today's terms, result in annual losses of approximately $700 million for producers and $1.8 billion for the Texas economy, based on USDA scenario analyses adjusted for inflation and assuming a 10% livestock morbidity rate and trade restrictions. These figures highlight the disproportionate economic vulnerability in comparison to direct public health risks. Beyond technical capacity, outcomes depend on reporting compliance, traveler awareness, and trust in genetic control; targeted One Health communication and community engagement are essential.

Control and Prevention Strategies

SIT remains the cornerstone of prevention. Its effectiveness stems from the monogamous mating behavior of females, ensuring sterile male releases can collapse populations [13]. The Panama barrier continues to function as a continental shield, with the Pacora sterile fly facility historically producing tens of millions of sterile flies weekly [3]. Plans for a new SIT facility in Texas represent an essential redundancy in case of regional incursions. SIT remains the cornerstone of prevention, but it is a resource-intensive approach. It requires steady funding, secure sterile-fly production, and reliable release logistics. These demands can strain fragile or low-resource settings, so redundant capacity is prudent.

Surveillance remains equally critical. Entomological trapping, coupled with veterinary and wildlife monitoring, enables early detection. Rapid responses, as seen in the Maryland case, are effective at excluding establishment. Climate change poses new challenges by expanding the ecological niche of *C. hominivorax*. Rising temperatures and altered precipitation may make parts of the southern United States increasingly suitable for survival [6,19]. Extreme weather conditions may also increase the likelihood of livestock wounds, providing new entry points. Anticipating these shifts through modeling will be essential for adapting control strategies [19]. International coordination is indispensable. The FAO, World Organization for Animal Health, and IAEA have all prioritized screwworm as a transboundary disease. Past multinational efforts eradicated the parasite from North Africa and Central America; current outbreaks underscore the need for renewed engagement and shared resources [15,20].

Future Directions and One Health Outlook

Integrated monitoring should draw on human clinical detection of unusual wound infestations, veterinary surveillance of livestock and pets, and wildlife monitoring. Entomological trap data should be combined with climate and ecological variables to model shifting distributions. Preparedness also requires institutional capacity: redundant SIT facilities, contingency stockpiles, harmonized protocols, and cross-border agreements. Global mobility of people and goods necessitates transnational approaches rather than national silos [21]. Research priorities include developing genetic suppression technologies, such as gene drives, which involve engineered genes that bias inheritance to reduce population viability. Preliminary studies suggest gene drives could target screwworm reproduction with high specificity, but ecological risks (e.g., unintended spread to nontarget species) and governance challenges (e.g., international regulatory frameworks) remain unresolved [22,23]. Additional priorities include advancing molecular diagnostics for earlier detection and refining climate-based predictive models [24]. Sociobehavioral research is also needed to optimize farmer compliance, traveler awareness, and community acceptance of SIT and emerging genetic tools [21,25].

Discussion

This narrative review synthesizes biological, historical, and contemporary evidence to contextualize the 2025 U.S. travel-associated human screwworm case within a One Health risk landscape. *C. hominivorax* remains a uniquely destructive ectoparasite due to its larvae's invasion of living tissue, producing rapidly progressive wounds in humans and animals and driving disproportionate agricultural losses when infestations reach livestock at scale [1,4,11,22]. Historical eradication in the United States through SIT, maintained by the Panama barrier, demonstrates that population suppression is feasible when biological insight, operational capacity, and political commitment align [10-13]. However, resurgence in Central America since 2023 and the 2025 Maryland case highlight that eradication success is fragile due to persistent regional endemicity and global mobility [8,16,18].

From a public-health standpoint, the immediate risk to the U.S. general population remains low without local transmission. Most imported human cases are isolated and resolve with prompt intervention [2,4,16]. By contrast, veterinary and agricultural risks are material: once established in suitable ecologies, screwworm populations can expand rapidly, with the highest morbidity around neonatal navels and surgical or traumatic wounds in livestock [10,23]. Economic analyses of historical incursions underscore the potential for substantial producer and broader macroeconomic losses if detection is delayed and emergency control is required [11,25]. Two cross-cutting pressures may elevate future risk. First, climate variability and long-term warming can expand ecological suitability and lengthen breeding seasons, potentially improving overwinter survival in currently marginal regions [6,26]. Second, globalization increases the movement of people, animals, and goods, multiplying introduction pathways arguments for embedding transportation and trade network analytics within routine risk assessment [7,9,24]. Operationally, resilience depends on redundant SIT capacity, sustained funding for the barrier program, and continuous entomological intelligence to enable fast, proportionate responses to incursions [3,12,13,20].

Emerging genetic control tools (e.g., gene drives or sex-bias systems) could, in principle, complement SIT, but their ecological externalities and transboundary implications necessitate rigorous risk assessment, international governance, and public engagement before any field deployment [14,15]. Advances in molecular tools, including genetics-based program enhancements, also hold promise for earlier, more specific detection and more efficient suppression operations [15,18]. Finally, effective prevention is not purely technical: acceptance of control strategies, adherence to livestock wound-care practices, and traveler awareness all require One Health-aligned risk communication and cross-sector coordination [21,24].

## Conclusions

The 2025 Maryland travel-associated screwworm case serves as a sentinel event, highlighting the persistent threat of *C. hominivorax* reintroduction from endemic regions amid rising global mobility. While human health risks in the United States remain low without local transmission, the potential for rapid population expansion in livestock poses significant agricultural and economic consequences. Climate change and globalization further amplify these risks by expanding ecological suitability and introduction pathways. Sustained protection hinges on integrating clinical, veterinary, and environmental surveillance with robust SIT capacity and international collaboration. Priority actions include funding continuity for the Panama barrier, redundant domestic SIT capacity, and a cross-border early-warning network linking veterinary, wildlife, and public health surveillance. Embedding these efforts within a One Health framework is essential to prevent *C. hominivorax* from reemerging as a domestic threat. Because introductions track the movement of people, animals, and goods, sustaining U.S. gains ultimately depends on regional control and international collaboration; eradication security is globally interdependent.
